# Is the motivation to quit smoking greater if the smoker is going to quit smoking of their own free will or when advised by a health professional?

**DOI:** 10.18332/tid/146961

**Published:** 2022-05-13

**Authors:** José I. de Granda-Orive, José Francisco Pascual-Lledó, Santos Asensio-Sánchez, Segismundo Solano-Reina, Marcos García-Rueda, Manuel Ángel Martínez-Muñiz, Lourdes Lázaro-Asegurado, Daniel Buljubasich, Susana Luhning, Rogelio Pendino, Isabel Cienfuegos-Agustín, Carlos A. Jiménez-Ruiz

**Affiliations:** 1 Respiratory Department, Hospital Universitario 12 de Octubre, Complutense University of Madrid, Madrid, Spain; 2 Respiratory Department, General University Hospital of Alicante, Alicante, Spain; 3 Respiratory Department, Hospital General Universitario Gregorio Marañón, Madrid, Spain; 4 Respiratory Department, Hospital Regional Universitario Carlos Haya de Málaga, Málaga, Spain; 5 Respiratory Department, Hospital Universitario San Agustín, Avilés, Spain; 6 Respiratory Department, Hospital Universitario de Burgos, Burgos, Spain; 7 Respiratory Department, Sanatorio Nuestra Señora del Rosario, Rosario, República Argentina; 8 Neumóloga, Centro Médico Humana, Córdoba, República Argentina; 9 Unidad Especializada de Tabaquismo de la Comunidad de Madrid, Hospital Clínico San Carlos, Madrid, Spain

**Keywords:** smoking, motivation scales, smoking cessation, volition

**Dear Editor,**

As a complement to a previous study by our group whose data and methodology have already been published^1,2^, we hypothesize that the motivation to quit smoking could be greater if the subject is going to quit smoking of their own free will (OFW) than if they are sent on the advice of a health professional.

The aim of the study was to check whether the degree of motivation to quit smoking is different depending on who refers the smoker to the smoking treatment consultation, considering three sources of remission (variable ‘referred by’): primary care (PC), medical specialist (OS) or by OFW.

For this analysis, the subjects finally included were 292 [72.1%; 155 women (53.1%); mean age 51.1 ± 11.0 years (range: 25–77)]. Ninety-nine subjects (33.9%) attended our smoking clinics on PC advice, 116 (29.7%) subjects on OS advice, and 77 subjects (26.4%) on OFW. We have used four motivational tests to quit smoking (MTQS): Richmond Test (RT), the Henri Mondor Paris Motivation Test (HMPMT), Khiwji-Watts test (KWT) and the visual analogue scale (VAS)^1^.

Supplementary file Table 1 shows the distribution of the three categories of the variable ‘referred by’ for all participants and by sex, and we found no significant differences. Table 1 shows the mean age values for all participants, by sex, and by each category of the variable ‘referred by’. There were no statistically significant differences between the mean ages of the different categories. Table 1 also shows the mean values of the scores of the MTQS according to the categories of the variable ‘referred by’, for all participants and by sex. Only in the HMPMT were there significant differences between PC versus OS, but this was only for men.

**Table 1:** Description of the quantitative variables for all participants, by sex, and by ‘referred by’

*Characteristics*	*All*	*Males*	*Females*	*p*
**Total**, n	292	137	155	
**Age** (years), mean ± SD (range)	51.1 ± 11.0 (25–77)	51.5 ± 11.1 (27–77)	50.7 ± 10.9 (25–76)	0.522
***Referred by***	***Primary Care***	***Other Specialties***	***Own Free-Will***	***p***
**Total**, n				
All	99	116	77	
Males	45	61	31	
Females	54	55	46	
**Age** (years), mean ± SD (range)				
All	50.0 ± 10.9 (29–77)	53.0 ± 10.9 (25–77)	48.7 ± 10.9 (26–72)	0.084
Males	51.4 ± 11.0 (29–77)	53.6 ± 10.6 (28–77)	47.8 ± 11.7 (27–66)	0.122
Females	48.9 ± 10.8 (29–70)	52.3 ± 11.3 (25–76)	50.9 ± 10.3 (26–72)	0.248
**Motivation scales** (scores), mean ± SD (range)				
**All**				
***RT***	7.9 ± 1.6 (3–10)	7.8 ± 1.5 (4–10)	8.2 ± 1.4 (5–10)	0.340
***HMPMT***	13.8 ± 2.4 (6–18)	12.6 ± 2.7 (3–18)	13.5 ± 2.9 (5–18)	0.003
***KWT***	11.6 ± 2.6 (5–15)	11.2 ± 2.6 (5–15)	11.7 ± 2.4 (7–15)	0.302
***VAS***	8.1 ± 1.8 (0–10)	7.7 ± 2.1 (0–10)	8.2 ± 1.7 (0–10)	
MD (SE) (95% CI)*		1.2 (0.4) (0.3–2.1)		
**Males**				
***RT***	7.9 ± 1.6 (3–10)	7.9 ± 1.5 (4–10)	8.5 ± 1.3 (6–10)	0.122
***HMPMT***	14.2 ± 2.3 (10–18)	12.7 ± 2.7 (7–18)	13.5 ± 3.1 (7–18)	0.021
***KWT***	11.3 ± 2.6 (7–15)	10.9 ± 2.6 (5–15)	12.3 ± 2.6 (7–15)	0.054
***VAS***	8.3 ± 1.9 (0–10)	7.9 ± 1.9 (2–10)	8.5 ± 1.6 (4–10)	0.216
MD (SE) (95% CI)*		1.5 (0.5) (0.2–2.8)		
**Females**				
***RT***	7.9 ± 1.7 (4–10)	7.8 ± 1.6 (4–10)	7.9 ± 1.4 (5–10)	0.890
***HMPMT***	13.4 ± 2.5 (6–18)	12.4 ± 2.8 (3–18)	13.5 ± 2.7 (5–18)	0.068
***KWT***	11.8 ± 2.7 (5–15)	11.5 ± 2.7 (5–15)	11.3 ± 2.3 (7–15)	0.475
***VAS***	8.0 ± 1.7 (3–10)	7.5 ± 2.3 (0–10)	8 ± 1.7 (0–10)	0.733

RT: Richmond Test. HMPMT: Henri Mondor Paris Motivation Test. KWT: Khiwji-Watts Test. VAS: Visual Analogue Scale. SD: standard deviation. MD: means differences. SE: standard error.

* Primary Care versus Other Specialties for HMPMT.

A previous study concluded that smoking cessation is motivated by concern for self-health and family’s health, family’s support, and social pressures^3^. In some studies, promptings by doctors were reported as being a reason for quitting by only 13% of respondents, and only one quarter of respondents received cessation-related awareness from their doctors^4^. It is known that personal willpower is an essential feature of the 5As model in ‘Treating Tobacco Use and Dependence’^5^, of which the first three As build towards willingness to quit and the last two As facilitate those willing to quit to take the final decision to quit^5^. This suggests how personal motivation that arises from within the individual is more likely to lead to successful cessation than when it arises externally^3^, but also, it is known, that a specific referral to a smoking cessation program can increase participation by patients^6,7^.

So, we cannot demonstrate differences in the scores of the analyzed smoking cessation motivation scales depending on who refers the subject. Subjects who attend smoking cessation clinics of their OFW do not have higher scores on the motivation questionnaires used when compared to those who attend on the advice of their PC or OS.

## Data Availability

Data sharing is not applicable to this article as no new data were created.
