# Supplementary material for: Is the motivation to quit smoking greater if the smoker is going to quit smoking of their own free will or when advised by a health professional?
Source: Tob Induc Dis. 2022 May 13;20:47. doi: 10.18332/tid/146961 (PMC9100290; doi:10.18332/tid/146961)
Supplement: Supplementary file 1 [file TID-20-47-s1.pdf]

Supplementary Table 1. Frequency distribution of the different categories of the variable 'referred by', for all participants and by sex (N=292)

	**All**	**Males**	**Females**	p
**Referred by**	n (%)	n (%)	n (%)	0.229
Primary Care	99 (33.9)	45 (32.8)	54 (34.8)	
Other Specialties	116 (39.7)	61 (44.5)	55 (35.5)	
Own Free-Will	77 (26.4)	31 (22.6)	46 (29.7)	

© 2022 de Granda-Orive J.I. et al.
